# Species- and site-specific circulating bacterial DNA in Subantarctic sentinel mussels *Aulacomya atra* and *Mytilus platensis*

**DOI:** 10.1038/s41598-022-13774-1

**Published:** 2022-06-10

**Authors:** Sophia Ferchiou, France Caza, Richard Villemur, Stéphane Betoulle, Yves St-Pierre

**Affiliations:** 1 INRS-Centre Armand-Frappier Santé Technologie, 531 Boul. des Prairies, Laval, QC H7V 1B7 Canada; 2 UMR-I 02 SEBIO Stress environnementaux et Biosurveillance des milieux aquatiques, Université Reims Champagne-Ardenne, Campus Moulin de la Housse, 51687 Reims, France

**Keywords:** Ecology, Microbial ecology

## Abstract

Impacts of climate changes are particularly severe in polar regions where warmer temperatures and reductions in sea-ice covers threaten the ecological integrity of marine coastal ecosystems. Because of their wide distribution and their ecological importance, mussels are currently used as sentinel organisms in monitoring programs of coastal ecosystems around the world. In the present study, we exploited the concept of liquid biopsy combined to a logistically friendly sampling method to study the hemolymphatic bacterial microbiome in two mussel species (*Aulacomya atra* and *Mytilus platensis*) in Kerguelen Islands, a remote Subantarctic volcanic archipelago. We found that the circulating microbiome signatures of both species differ significantly even though their share the same mussel beds. We also found that the microbiome differs significantly between sampling sites, often correlating with the particularity of the ecosystem. Predictive models also revealed that both species have distinct functional microbiota, and that the circulating microbiome of *Aulacomya atra* was more sensitive to changes induced by acute thermal stress when compared to *Mytilus platensis.* Taken together, our study suggests that defining circulating microbiome is a useful tool to assess the health status of marine ecosystems and to better understand the interactions between the sentinel species and their habitat.

## Introduction

Climate changes are known to alter biodiversity at a global scale^1,2^. This is particularly true for Subantarctic regions, which have undergone significant changes in recent years due to global warming^3–5^. Sea surface temperatures in the Western Antarctic Peninsula have increased by ~ 1 °C since the industrial revolution and is expected to accelerate with the constant augmentation of global CO_2_ concentrations, as suggested by predictive models^6,7^. The consequences of the global warming include alterations of the interactions between hosts and their associated microbiomes (*i.e.*, assemblages of microorganisms)^8–10^. Such dysbiosis could not only affect marine ecosystems per se, but also favors the emergence and proliferation of opportunistic pathogens^11,12^. Emerging infectious disease outbreaks in marine ecosystems would not only impact directly on the host populations but can also lead to mass mortalities of keystone species^9,13^. This is particularly true for sedentary and filtrating marine organisms such as mussels and oysters that are more prone to frequent, episodic mortality events during heat waves in summer^14–16^. Moreover, bivalves, which play a central role in an ecosystem integrity, are the most widely used sentinel species to monitor coastal marine environments as they are sedentary and have high filtering capacities leading to accumulation of pathogens and pollutants^17,18^. Their high filtering activity links them to their surrounding environment. Many marine mussel-based biomarkers have thus been developed over the years for monitoring environmental quality and for assessing environmental variations, particularly in remote areas such as polar regions^17,19,20^.

The development of ethical (non-lethal) and logistically friendly sampling methods combined with the concept of liquid biopsy offers, however, a new window of opportunity to develop highly sensitive and predictable biomarkers^21^. Liquid biopsy, combined with high throughput DNA sequencing of circulating plasmatic DNA fragments, is commonly used in the biomedical field for decision-making in patients with cancer^22^. More recently, it has also been shown to be a useful mean to measure the presence of DNA fragments of non-self origin even in absence of overt diseases^23^. This has led to a novel concept, the “circulating microbiome DNA” (cmDNA)^24^. In contrast to tissue-specific microbiome analysis, characterization of the cmDNA allows to obtain a signature that reflects bacterial populations derived from classical niches (such as the gut, skin, oral cavity, etc.). This is also true in sentinel species, such as bivalves, which hemolymphatic cmDNA contains a rich and diverse microbiome and can be used as biomarkers to reflect host fitness and health status^25–29^. In fact, characterization of microbiomes in general has become an important tool to measure the impact of environmental and anthropogenic stressors in health and resilience of marine coastal ecosystems^29^. For example, in the case of *M. galloprovincialis*, in vivo exposure to polystyrene nanoplastics significantly affects different hemolymph immune parameters and a shift the ciruclating microbiome^27^.

The coastal marine ecosystems of the Subantarctic Islands of Kerguelen (49°26′S, 69°50′E) are inhabited by two mussel species: the Subantarctic population of blue mussels, *Mytilus platensis* (*M. platensis*), and the ribbed mussel *Aulacomya atra* (*A. atra*). The isolated archipelago of Kerguelen is part of an oceanic submerged plateau that was built 35 million years ago following continuous volcanic activity. This archipelago is home to a maritime nature reserve classified as a UNESCO World Heritage Site since 2019. It is also a central hub for research on marine biodiversity in the Southern Ocean^30^. Recent observations indicate that Kerguelen Plateau is also characterized by the submarine gas emission that may represents active volcanic activity associated with cold seeps and hydrothermal vents^31^. This makes Kerguelen a unique site to measure the impact of climate changes on marine coastal habitats. In the present work, we report our findings of the first study of the hemolymphatic circulating microbiome collected in the two mussel species that inhabit mixed mussel beds located at different sites of the Kerguelen Islands.

## Results

### Species-dependent variations in bacterial communities

**Figure 1:**
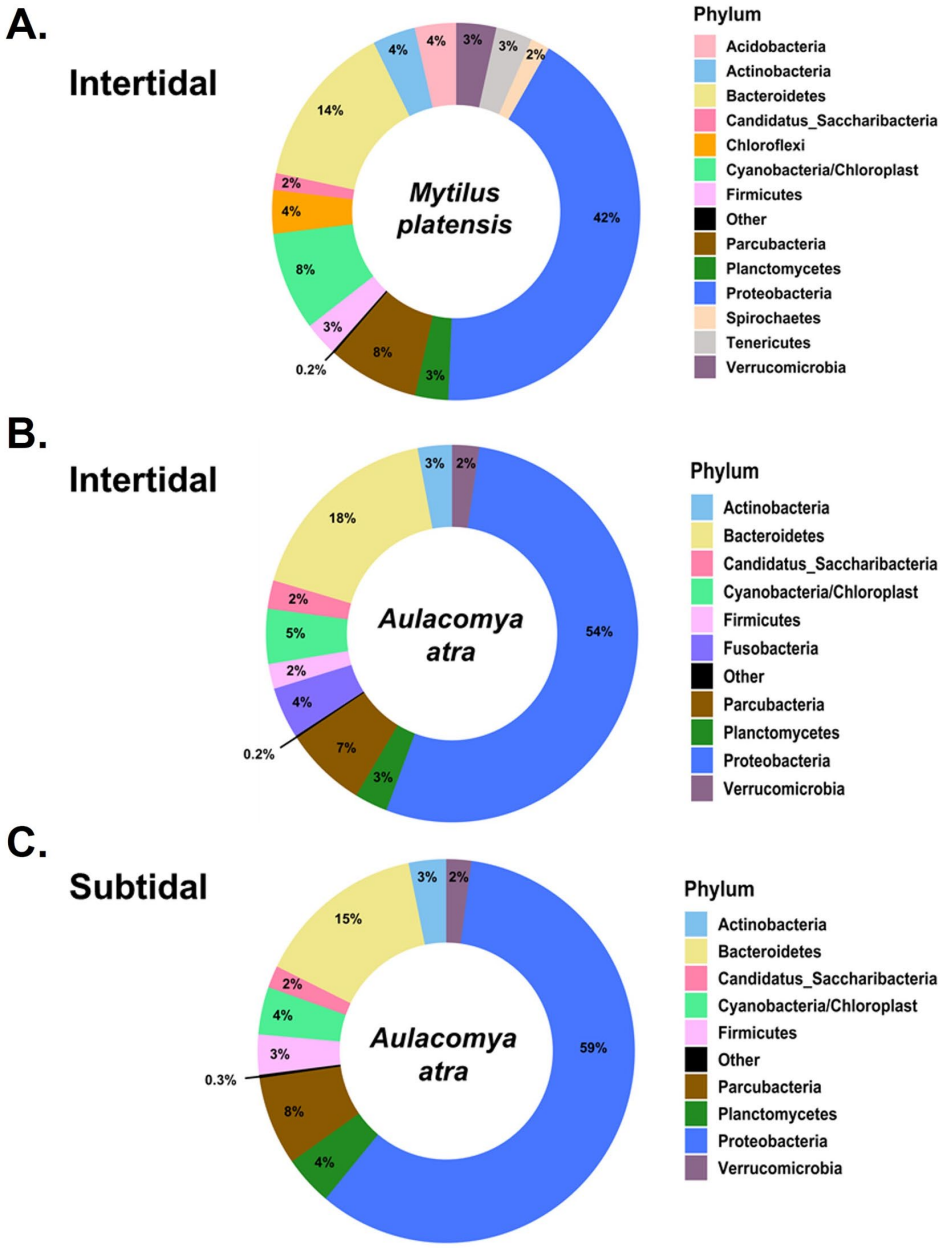
Phylum-level analysis of hemolymphatic bacterial DNA. (**A**) *M. platensis* and (**B**) *A. atra* collected in intertidal zones. (**C**) of *A. atra* collected in subtidal zones. Phylum with a relative abundance of ≤ 1.5% are represented as "Other".

A total of 150 samples of *M. platensis* (all from the intertidal zone) and *A. atra* (intertidal and subtidal) were analyzed. For each sample, the cmDNA was profiled by sequencing the 16S rRNA gene amplicons of the V3–V4 hypervariable region. A total of 6,489,570 paired-end sequences passed quality filtering (43,264 ± 27,652 per sample). Amplicon sequence variants (ASVs) were generated from 21,389 high-quality reads. Overall, the relative abundance of bacterial phyla in both mussel species were dominated by *Proteobacteria* (56.0% and 41.6% for *A. atra* and *M. platensis* respectively), *Bacteroidetes* (14.8% and 14.2% respectively), and *Parcubacteria* (7.2% and 8.3% respectively), which accounted for more than 60% of all reads (Fig. 1). This composition is consistent with a recent metagenomic study showing that bacteria enriched in seawater in polar regions were mostly *Proteobacteria*, *Actinobacteria*, *Bacteroidetes* and *Parcubacteria*^32^. Multivariate analysis (PERMANOVA) on a weighted UniFrac showed, however, that the global composition of the microbiome was significantly different between *A. atra* and *M. platensis* (weighted UniFrac PERMANOVA, F_(1, 154)_ = 5.02, *p* < 0.001). A case in point at the phylum level is the presence of *Acidobacteria* (4%), *Chloroflexi* (4%), *Spirochaetes* (2%), *Tenericutes* (3%), and *Verrucomicrobia* (3%) in *M. platensis* but not in *A. atra*. In contrast, *Fusobacteria* (4%) were more abundant in *A. atra* in the intertidal zone. This phylum is commonly found in marine sediment environments but has been reported to constitute the major phylum in oil-contaminated anaerobic niche of seawater following the deep-water horizon oil spill and other marine reservoirs^33,34^. Interestingly, the highest abundance of *Fusobacteria* was detected at Port-aux-Français, the capital settlement of Kerguelen Islands and the only site with active port activity (Supplementary Figure S1). Furthermore, no significant differences in the composition of the microbiome were found in *A. atra* between subtidal and intertidal zones (weighted UniFrac PERMANOVA, F(1, 88) = 1.49, *p* = 0.103)).

### Site-dependent beta diversity analysis

**Figure 2:**
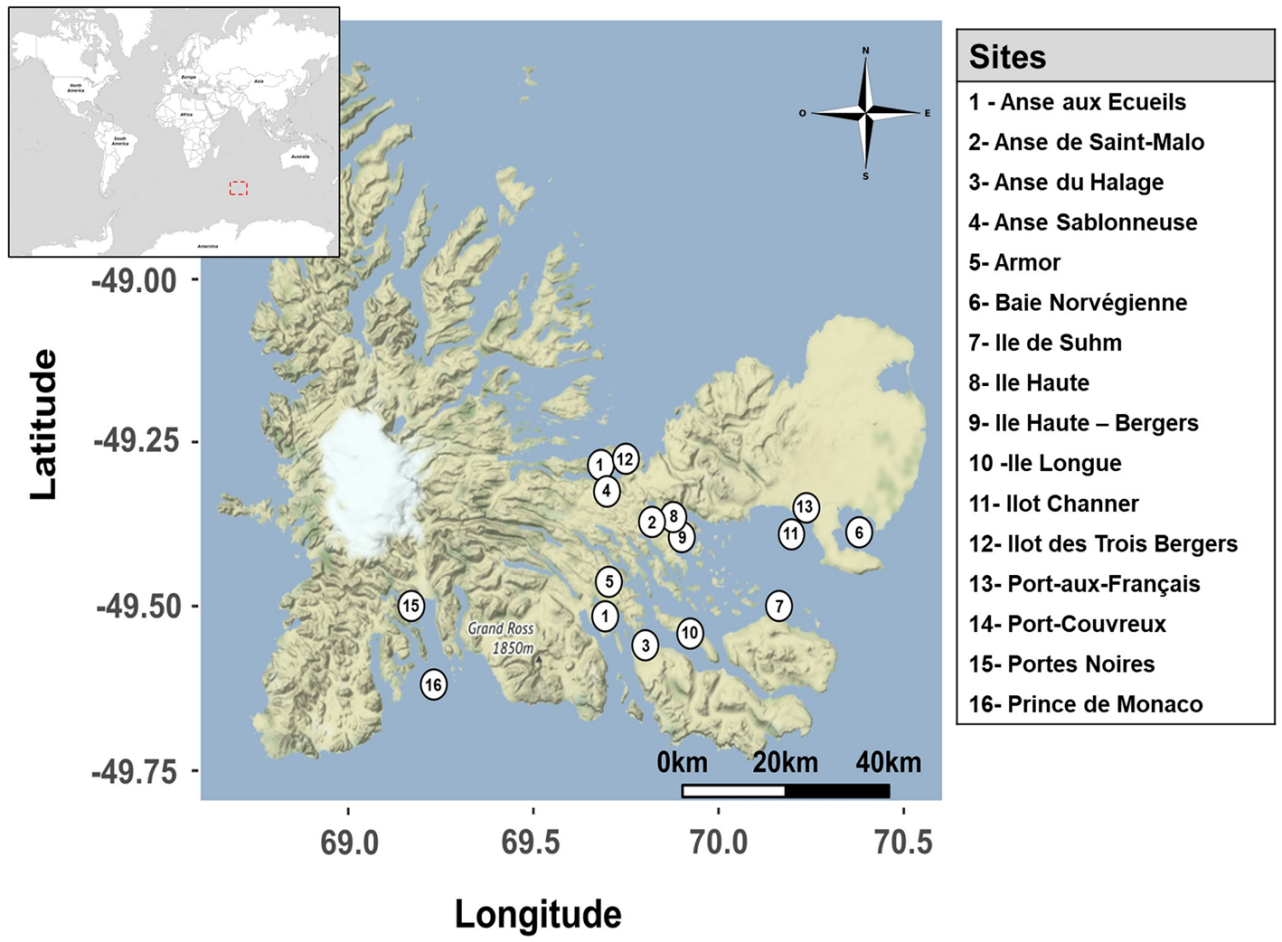
Map of the French Subantarctic Kerguelen Islands showing the sampling sites. The map was generated with the ggmap package in R programming language (R version 4.0.3, R Core Team^88^).

**Figure 3:**
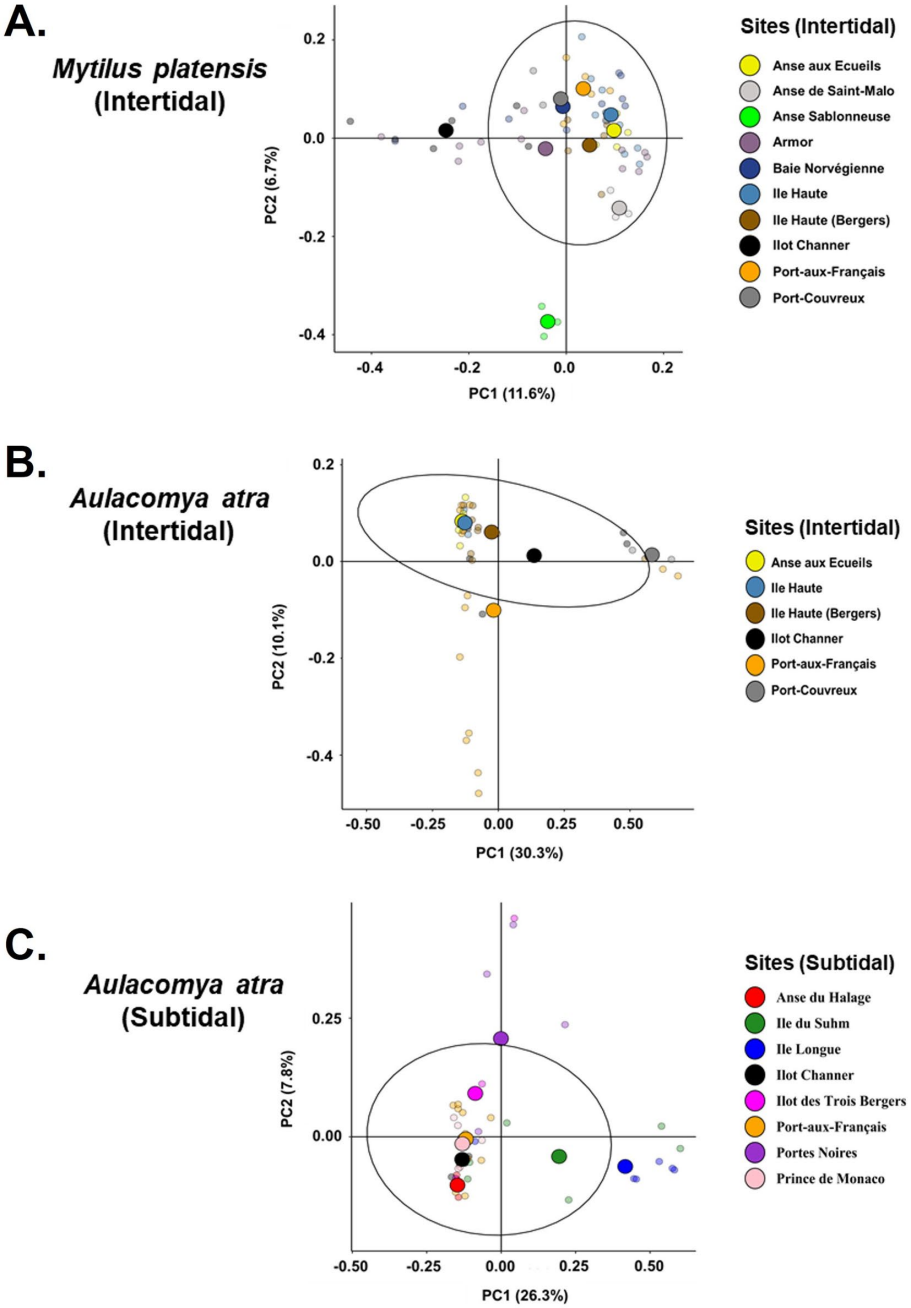
Principal Coordinates Analysis (PCoA) of bacterial DNA bacterial communities at different sites. Weighted UniFrac-based of in samples collected in *M. platensis* (**A**), *A. atra* in intertidal (**B**) and subtidal (**C**) zones. Centroids for each site are illustrated by larger circles. Black ellipses represent 90% confidence interval.

We next examined spatial variations in the cmDNA profiles of both mussel species. For this, we collected samples from 16 sites, including 5 mixed mussel beds (Port-aux-Français, Port-Couvreux, Île Haute, Île Haute-Baie des Bergers, and Anse-aux-Écueils) and 5 subtidal sites where samples of *A. atra* were collected (Fig. 2). To compare β-diversity between sampling sites, principal coordinate analysis (PCoA) based on the weighted UniFrac distance was carried out for both species (Fig. 3). Multivariate analysis revealed a significant separation of samples according to sampling sites. An ADONIS on weighted UniFrac analysis showed a significant difference according to sampling sites (*p* < 0.001) for both species. For *M. platensis*, distances to group centroids were significantly different (Permutest, F_(10, 55)_ = 4.83, *p* = 0.002) in samples collected at Anse de Saint-Malo and Anse Sablonneuse (Fig. 3A). In the case of A. *atra*, the betadisper analysis highlights four distinct microbial sites where the community structure was significantly higher (Permutest, F_(13, 75)_ = 3.32, *p* = 0.002); Port-Couvreux (intertidal), Îlot Channer (intertidal), Anse-aux-Écueils (intertidal) and Anse-du-Halage (subtidal) showed a strong separation with other sites.

### Site-dependent alpha diversity analysis

**Figure 4:**
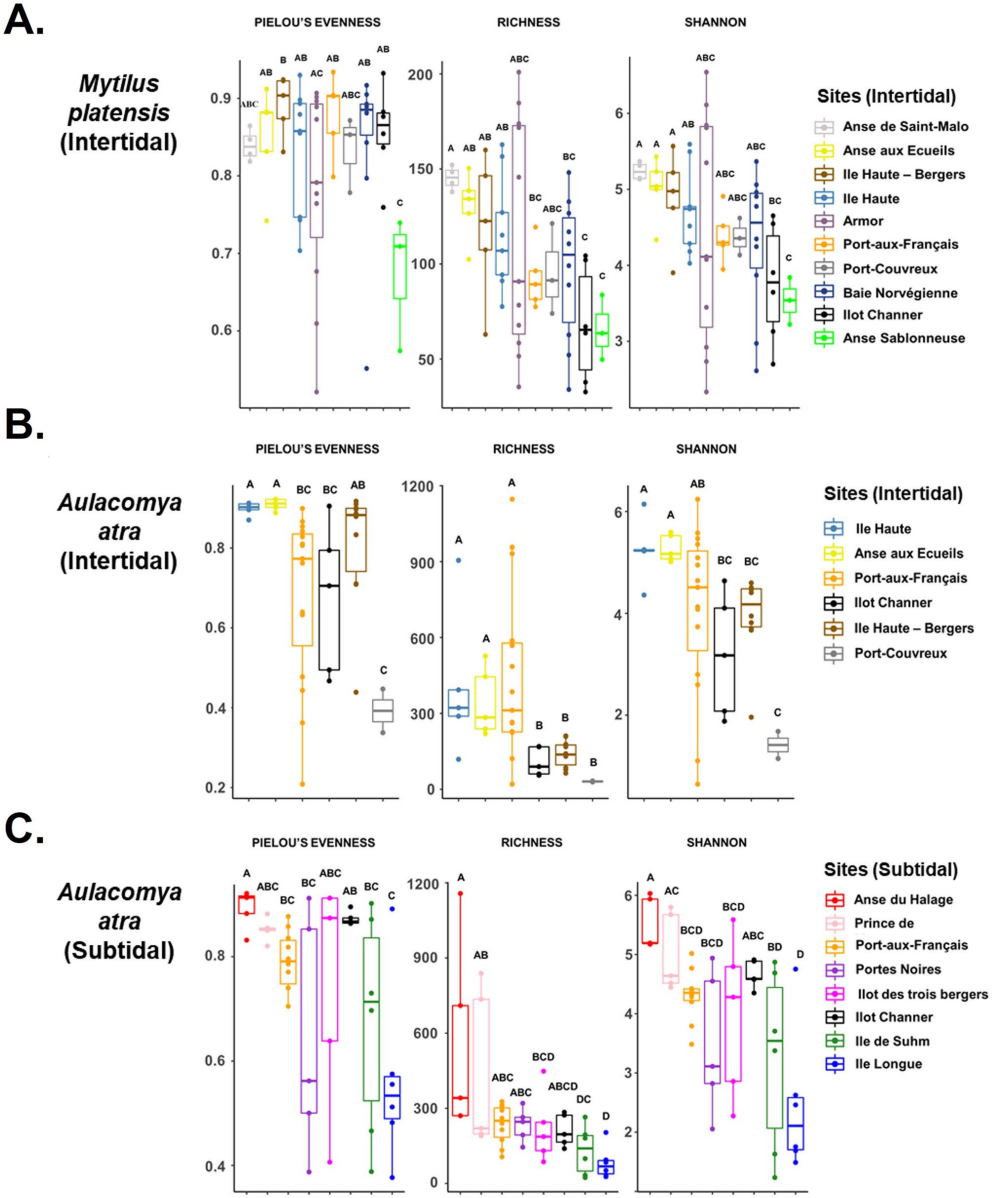
Alpha diversity analysis. Box plots of alpha diversity of (**A**) *M. platensis* collected in intertidal zone and *A. atra* in intertidal (**B**) and subtidal (**C**) zones. Species evenness observed richness and Shannon diversity indexes were calculated for each site. Significant *p* values (*p* < 0.05) were obtained using permutation test as a non-parametric test following by a post-hoc pairwise comparisons.

We next compared the alpha diversity for each site using three diversity indices including richness, Shannon index, and Pielou’s evenness. Globally, we found no significant differences in the cmDNA for both mussel species collected during two successive campaigns at Kerguelen (2017 and 2018) (Supplementary Figure S2). A comparative analysis of the alpha diversity according to sites revealed, however, significant differences between sites for both species (Fig. 4). In the case of *M. platensis*, the richness estimator showed a significant variation (χ^2^ = 18.977, *p* = 0.0401, *df* = 10). The lowest richness value was observed at Anse Sablonneuse (65.7 ± 9.9), a mussel bed occurring in mid to lower shore sand. In the case of *A. atra*, richness estimator (χ^2^ = 38.779, *p* < 0.001), Shannon index (χ^2^ = 37.996, *p* < 0.001) and Pielou’s evenness (χ^2^ = 36.647, *p* < 0.001) varied greatly between sites for both intertidal and subtidal zones. Lowest alpha diversity indices in *A. atra* sites were observed at Port-Couvreux (intertidal) and Île Longue (subtidal).

### Site-dependent variations at the genus level

**Figure 5:**
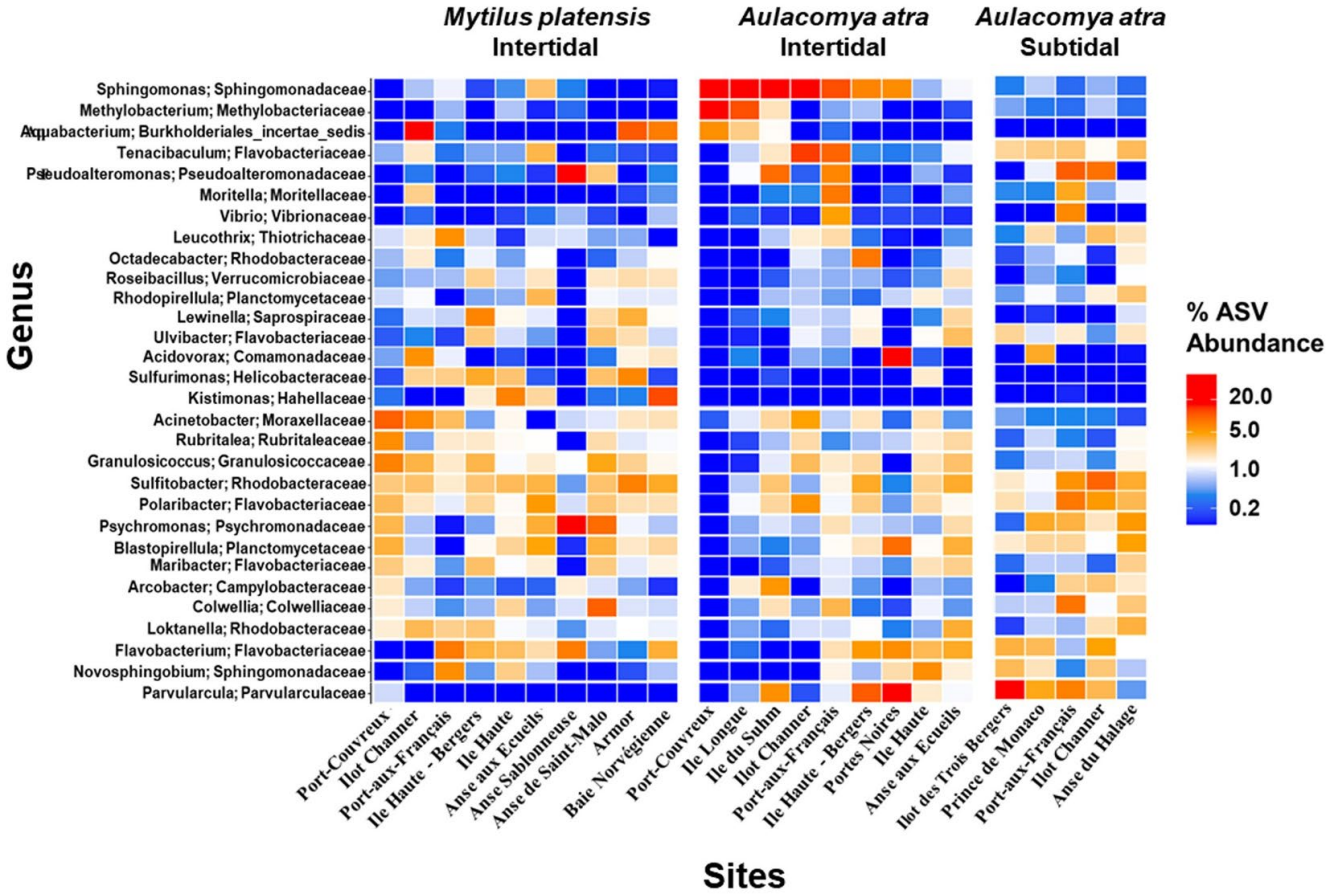
Heatmaps showing relative abundance (%) of the top 30 bacterial genera of hemolymphatic microbiota between in both mussel species. Red colors indicate higher abundance and blue colors indicate lower abundances.

Differences between sites for both species were further documented at the genus level, focusing first on the top 30 genus, which represent 32% of all genera. Among the notable findings, we found strong abundance of *Pseudoalteromonas* and *Psychromonas* in *M. platensis* collected at the Anse Sablonneuse (Fig. 5). The cmDNA of *M. platensis*, but not *A. atra*, collected in the intertidal zone of the Îlot Channer, an islet formed of basaltic rocks surrounded by a belt of large brown algae (*Durvillea* and *Macrocystis*), was dominated by *Aquabacterium*, a common bacteria found within kelps^35^. In the case of *A. atra*, we found a relative abundance of *Sphingomonas* at several sites, including Port-Couvreux, Ile Longue, Île du Suhm and Portes-Noires which accounted for 31.5–57.7% of total reads (Supplementary Table S1). This genus dominance could be attributed to the significant occurrence of kelp (*Macrocystis pyrifera*) at these sampling sites. This is in agreement with Florez et al.^36^ who reported that epiphytic bacterial communities in macroalgae are associated with *Sphingomonadales*/*Sphingomonas* are known to degrade alginate as carbon source^37,38^. It was also worth noting that *A. atra* collected at the intertidal zone of the fjord of Portes Noires showed quite a unique abundance of *Acidovorax, of* which several species are phytopathogenic*,* and *Parvularcula,* compared to any other sites or to *M. platensis*. Interestingly, abundance of *Vibrio* was higher in *A. atra* in both intertidal and subtidal mussels of Port-aux-Français.

The differences between sites were further studied at the genus level by focusing this time on the phyla that differed between both species. The most significant difference between *M. platensis* and *A. atra* was observed at Port-Couvreux (Supplementary Figure S3). We also found that bacterial DNA from the genus *Kistimonas*, a member of the *Hahellacae* family of *Proteobacteria*, was not detected in *A. atra* at any site but was present in *M. platensis* at Port-Couvreux, Île Haute, Île Haute-Baie du Berger, and Anse-aux-Écueils. Bacterial DNA from the genus *Sulforimonas* was also commonly found in *M. platensis* at several sites but rarely in *A. atra*. Overall, these results showed that both mussel species have distinct circulating microbiome profiles that vary according to the geographic locations of the mussel beds. Given the fact that season changes may play a major role in shaping bacterial community structure in bivalves, further investigations are needed to compare seasonal variability in the microbiome between both mussel species^39,40^.

Environmental microbiota can also be influenced by anthropogenic sources. This could explain why the relative abundance of bacteria of the *Vibrio* genus were more abundant at Port-aux-Français (in both intertidal and subtidal zones) in *A. atra* than at other sampling sites (Supplementary Table S1). LEfSe analysis indeed revealed that *Vibrio* was the top genus-level biomarkers that distinguished Port-aux-Français from all other sites (Supplementary Table S2). This finding is consistent with a previous study showing that anthropogenic impacts are higher at Port-aux-Français when compared to other sites^41^. In Mediterranean Sea, the proliferation of opportunistic pathogens affiliated to *Vibrio spp.* is suspected to be a major factor in disease outbreaks in bivalve species^42,43^. Moreover, *Vibrio spp.* encompasses a variety of pathogens whose presence within bivalve tissues and interactions with their immune system are well documented^44^. Taken together, our results indicate that hemolymph cmDNA profiles reflect the local environment and the impacts of anthropogenic stressors.

### Functional analysis of the cmDNA

**Figure 6:**
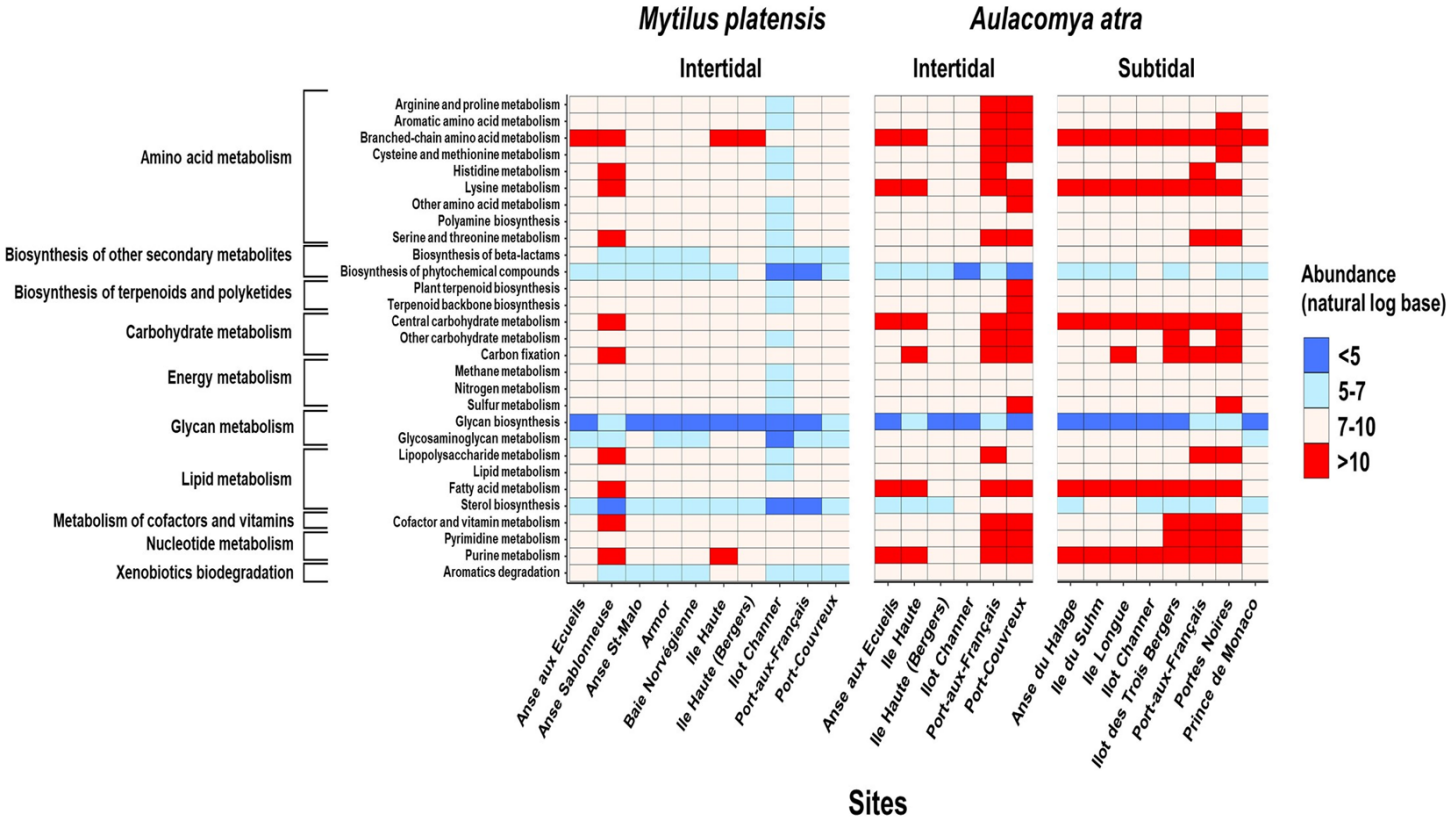
Predictive functional analysis. Heatmaps based on the main KEGG pathways predicted at the different sampling sites. Relative abundance (natural logarithm base) of metabolic pathways based on 16S rRNA data is shown for both mussel species. Enrichments correspond to the positive values (red color) while depletions correspond to negative values (blue).

Predictive functional analysis from 16 s rRNA profiling is becoming a common tool to link the abundance of specific taxa with metabolic profiles^45–47^. To further explore the distinctive traits that distinguish the circulating microbiome of mussels at different sites, we studied its functional content predicted from the KEGG database using Piphillin tool. Our findings confirmed that the bacterial pathways varied significantly between *A. atra* and *M. platensis* and between sites (Fig. 6). A case in point is the abundance of the pathways involved in xenobiotics degradation, carbohydrate metabolism and amino acid metabolism in the hemolymph of *A. atra* at Port-Couvreux.

### Effect of thermal stress on cmDNA

**Figure 7:**
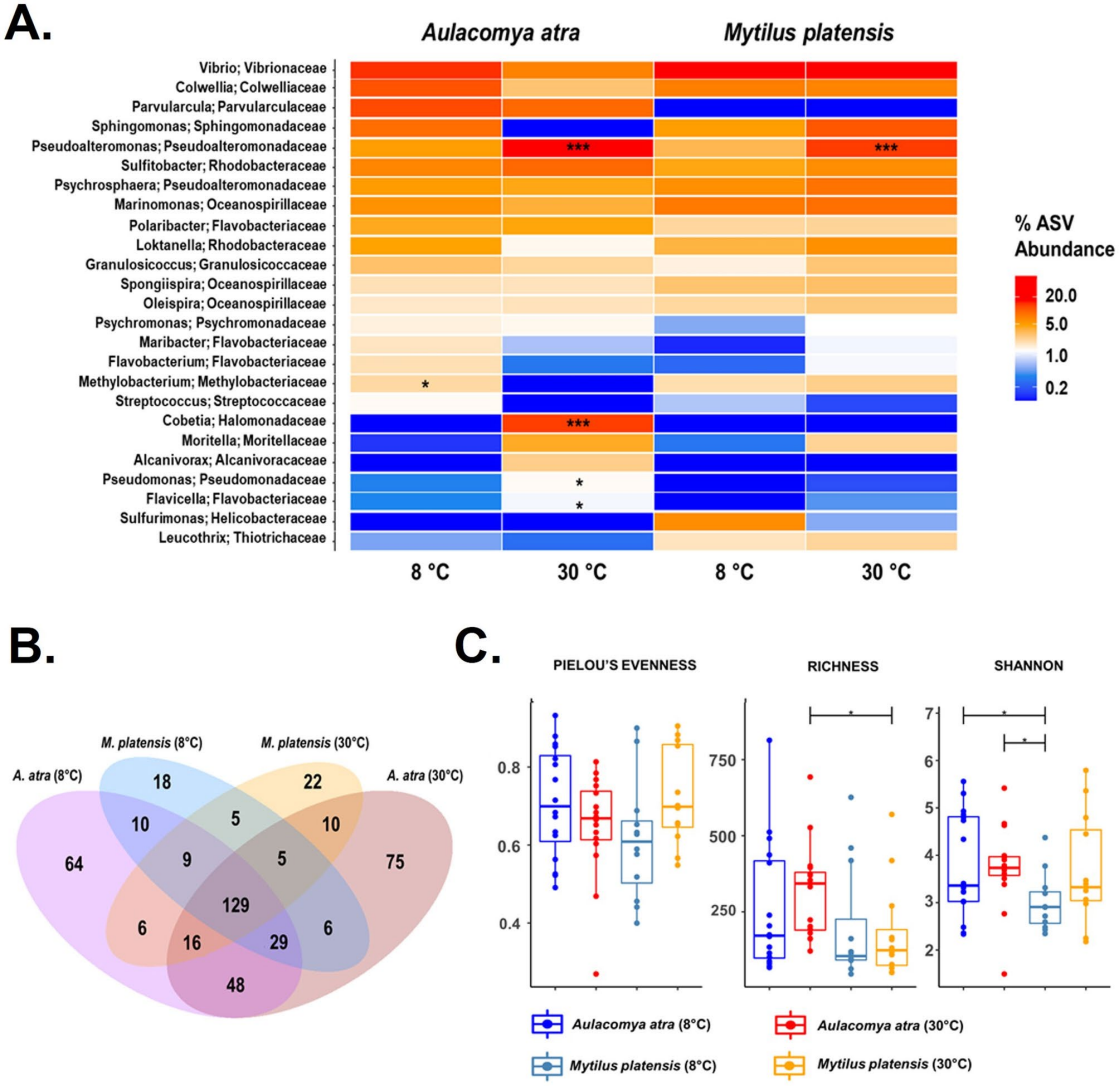
Changes in bacterial cmDNA profiles in mussels following an acute thermal stress. (**A**) Heatmap outlining the presence and the relative abundance (%) of the top 30 bacterial genera of at different temperatures. The data were generated from 8 345 ASVs. Red colors indicate higher abundance and blue colors indicate lower abundances. Stars indicate taxa that were significantly different from four groups, as determined by LDA Effect Size analysis. (**B**) A Venn diagram showing the number of unique and shared bacterial genera in both species at 8 °C and 30 °C. (**C**) Alpha diversity of *A. atra* and *M. platensis* hemolymphatic cmDNA at 8 °C and 30 °C. Species evenness, observed richness and Shannon diversity indexes were calculated for each group. Note: *0.01 < *p* ≤ 0.05; **0.001 < *p* ≤ 0.01; ***p ≤ 0.001.

Environmental conditions, particularly temperature, are known to affect the hemolymphatic microbiome in many marine invertebrates^26,48,49^. In fact, alteration of microbiota by thermal stress can induce mortality in some organisms^50^. To determine whether the circulating microbiome of both species was modulated equally to thermal stress, both mussel species was subjected to an acute thermal stress under laboratory conditions. Our results showed that *Pseudoalteromonas spp.*, a bacterial genus found in the bacterioplankton in Antarctica^51^, was the most abundant genus obtained in both mussel specimens and its abundance increased significantly (*p* < 0.001) in both mussels exposed at a higher temperature (30 °C) (Fig. 7A, Supplementary Table S3). A significant increase in *Cobetia*, a facultative psychrotrophic bacteria that can grow at temperature ranging from 0 to 40 °C^52^, was also seen in *A. atra* but not in *M. platensis*. A Venn diagram showed that the hemolymphatic cmDNA in *A. atra* harbored 3–4 times more genera that that of *M. platensis*, suggesting a higher diversity (Fig. 7B). The higher diversity found in *A. atra* was confirmed upon analysis of richness and Shannon indices (Fig. 7C). Taken together, these results suggest that both mussel species react differently to thermal stress, consistent with results reported in an experimental model of acute thermal stress^21^.

## Discussion

In the present work, using hemolymphatic liquid biopsies, we compared the cmDNA of two mussel species that cohabit the coastal ecosystems of Kerguelen. More specifically, we found that: 1) The cmDNA bacterial profiles of both species differ significantly; 2) differences were site-dependent; 3) both species had distinct functional microbiota, and 4) the circulating bacterial microbiome of *A. atra* was more sensitive to changes induced by an experimental model of thermal stress. Overall, this study shows that both mussel species of Kerguelen Islands have distinctive bacterial cmDNA signatures that can be used for long term monitoring of the impact of climate changes on marine coastal ecosystems. We also show that defining the bacterial cmDNA signature in sentinel species is compatible with a minimally invasive, ethical and logistically friendly sampling method adapted for research in remote regions such as Kerguelen Islands.

Our study has revealed that hemolymphatic bacterial DNA in mussels are species-specific, even though both mussel species co-habit intimately in mussel beds of Kerguelen. These results suggest the existence of a selective bacterial retention between the two mussel species. Similar conclusions have been reported recently by Weingarten et al.^53^ who studied the gut microbiota of four different freshwater *Unionidae* mussels. Several non-exclusive hypotheses can be formulated to explain these findings. First, this could be related to intrinsic characteristics of filtration between species^54^. For instance, *M. platensis* is characterized by longer labial palps allowing to capture and reject finer material in comparison with other bivalves and have higher filtration rates than *A. atra*^55,56^. It has also been suggested that the circulating microbiome is not just dependent of particles ingestion but may be impacted by the interactions between environmental microbiota and host gills for selectively retaining certain taxa. Indeed, it has been demonstrated that ctenidia (*i.e.,* paired gills) are responsible for particle sorting such as plankton, organic detritus and bacteria. Some studies have shown that picoplankton (0.2–2 µm) represents a significant contribution to the mussel diet and their ingestion was found to be cleared at higher rates than bacteria (~ 0.6 µm in size) by *M. platensis*^57–59^. This suggests that factors other than size affect capture of particles by ctenidia of bivalves, such as availability of the seston, might come into play. Marine aggregates (1–500 µm) may also contribute to enhance the uptake of picoplankton and bacteria^60,61^. Particle selection and ingestion in filter-feeding bivalves may thus depend on differential morphology of the ctenidia^54,62^. Particle discrimination can also be influenced by interactions between chemical constituents of the cohesive mucous of pallial organs and surface carbohydrates of captured particles^59,63^. Differences between microbiota in bivalves can also be modulated by the mucosal immunity^64–66^. Bivalve pallial mucus contains agglutinins that can interact with several bacterial species. It may also depends to immune humoral factors, such as antimicrobial peptides, that play a central role in defense, most notably in the extrapallial compartment where enriched nutrients such as proteins and polysaccharides that promote microbial proliferation^67^. Another immune mechanism that may explain the differential microbiome signature is the selective killing of bacteria bacteriophages^65^. For instance, two phages isolated in Pacific oyster larvae provide a non–host-derived immunity against pathogenic *V. coralliilyticus*^68^. Bacteriophage adherence to bivalve mucosal surfaces is central in this symbiotic relationship. Clearly, further comparative studies between *M. platensis* and *A. atra* are required to address this question.

Our data highlights that cmDNA bacterial profiles are site-specific and may provide critical information on the surrounding marine ecosystems. A good example is the presence sulfur-oxidizing bacteria (SOB) (e.g., *Sulfitobacter spp.* and *Sulfurimonas spp.*), which represent a significant part in hemolymph microbiota in both mussel species collected at different sites (Supplementary Table S1). SOB represent a large part of microbial communities in marine surface sediments where they play a key role in element cycling^69,70^. Coastal ecosystems around the Kerguelen islands are characterized by high wind speeds and strong tidal currents causing sediment dispersion along the coast^71–73^. In addition to symbionts, the region around the Kerguelen Plateau is characterized by the presence of cold seeps and hydrothermal vents emitting chemical-rich fluids^31^. It is likely that symbiotic SOB are present in the gills of the two mussels and contribute to provide organic carbon as energy source to the host by consuming sulfide from the Subantarctic vents. Another example of how the circulating microbiome can provide clues on local environmental conditions is the detection of DNA derived from thermophilic bacteria, such as *Geobacillus spp.* and *Thermomonas spp.*, which were found in *A. atra* collected at Îlot-des-Trois-Bergers and Prince-de-Monaco (Supplementary Table S1). Both sites are located near geothermally heated habitats^74^. High abundance of kelps could also modulate the circulating microbiome signature. The coastlines of Kerguelen Islands are occupied by highly productive giant kelp beds forming undersea forests in hard bottom. Giving the fact that numerous strains of the genus *Sphingomonas* can decompose algal polysaccharides and be a dominant bacterium from a marine oligotrophic environment^38,75^, it is therefore not surprising to find a great abundance of this genus at sampling sites characterized by kelp forests. Taken together, these results illustrate the potential of defining the circulating microbiome signature in mussels for long-term monitoring of environmental changes occurring in coastal marine ecosystems.

Intertidal mussels are exposed to sea surface and air temperature variations reflecting the solar exposition, the wind speed, the sea level pressure, humidity index and the dual tides in Kerguelen^76–78^. In 2018, according to the PROTEKER’s registries from their temperature loggers at Îlot Channer site at a depth of 5 m, the temperature varied from 2.7 to 9.5 °C, where *A. atra* is found^79^. The situation is of course quite different in the intertidal zones where temperature changes is subjected to high variations within a single day and between tides. Considering the growth rate of some bacteria, it is logical to expect a microbiome shift after a thermal stress as we did observe in our experimental model of thermal stress. Such effect of thermal stress on the cmDNA has been documented in the past for bivalves^26,40,48^. However, this effect was more pronounced in *A. atra* when compared to *M. platensis*, as shown by the significant increase of the bacteria of the *Cobetia* genus in *A. atra*. These bacteria can grow at temperature up to 40 °C^52^. In addition, it is also important to pinpoint that the microbiome signature in mussels will also be affected by seasonal variations.

Overall, our data with the circulating microbiome suggests that *A. atra* is more sensitive to thermal stress when compared to *M. platensis*. These results are consistent with previous studies comparing the hemolymphatic component of both species in response to thermal stress^80^. Whether such this plays a role in its progressive disappearance for the profit of *Mytilus spp.* in many marine ecosystems is unclear now^81^. Clearly, monitoring of mixed beds may represent a unique opportunity to study the effect of climate change on marine coastal ecosystems in the Southern hemisphere, a particularly sensitive region to climate change.

## Materials and methods

### Sample collection

*A. atra* and *M. platensis* specimens (55–70 mm length) were collected at 16 sites of the French sub-Antarctic Kerguelen Islands during the 2017 and 2018 Summer campaigns (Fig. 2). Liquid biopsy samples were collected from samples collected in both intertidal or subtidal (~ 5 m depth) zones and immediately processed (within an hour) on site as previously described^21^. Briefly, intravalvular liquid was removed with the tip of a knife and hemolymph withdrawn from the adductor muscle using a syringe fitted with a 21-gauge needle. Samples were immediately transferred into 1.5 mL sterile Eppendorf tube and centrifuged on site for 3 min at 3000×*g* at ambient temperature using a portable, battery-operated TOMY Multi Spin centrifuge (TOMY, Japan). Samples were pooled to eliminate individual differences and to minimize small-scale variability^25,82^. After centrifugation, cell pellets were dispersed gently and a 50 µL aliquot was applied on individual discs of Whatman 903™ FTA® cards (Sigma-Aldrich, Oakville, ON, Canada). After a 30 min drying period at ambient temperature, individual cards were kept in zip-sealed sampling plastic bags containing one small desiccant. Ethanol was used to disinfect handling equipment, and gloves were always worn during the procedure to prevent contamination from human hands.

### Thermal stress experiments

Adult specimens of *M. platensis* and *A. atra* were collected on the intertidal rocky shores of Port-aux-Français (49°21′4.682″ S, 70°13′22.496″ E) at Kerguelen Islands in November and December 2018. Mussels were transported to laboratory within the hour and immediately transferred in a temperature-controlled (8 °C) aerated aquarium containing filtered recirculating seawater maintained on a 12 h:12 h light/dark cycle for at least 24 h. Mussels were placed in a 30 °C seawater recipient for 90 min. This acute stress model is commonly used in other studies to investigate the effect of temperature stress between mussels congeners^80,83–85^. Controls included mussels incubated for the same period of time at 8 °C. After 90 min, hemolymph samples were immediately processed as described above and 70 µL of hemolymph was spotted on FTA cards kept in sample bags with a desiccant.

### Preprocessing and sequencing

Individual discs were cut from the FTA cards using a sterile 5.0 mm single round hole punch and total DNA isolated using the QIAamp DNA Investigator Kit (Qiagen, Hilden, Germany) according to the manufacturer’s protocol. Quantification of DNA was carried out in duplicate using Quant-iT™ PicoGreen® dsDNA detection kit (Molecular Probes, Eugene OR). Amplification of the 16S ribosomal RNA (rRNA) gene amplifications and 16S gene amplicon sequencing for all DNA samples were performed at Centre d'expertise et de services Génome Québec (Montréal, QC, Canada) using the universal primers 341F (5’-CCTACGGGNGGCWGCAG-3’) and 805R (5’-GACTACHVGGGTATCTAATCC-3’)^86^. Sequence libraries were prepared by Génome Québec with TruSeq® DNA Library Prep Kit (Illumina, San Diego, CA, USA) and quantified using KAPA Library Quantification Kit for Illumina platforms (Kapa Biosystems). Paired-end sequences were generated on a MiSeq platform PE300 (Illumina Corporation, San Diego, CA, USA) with the MiSeq Reagent Kit v3 600 cycles (Illumina, San Diego, CA, USA).

### Bioinformatic analysis

Illumina sequence data (FASTQ files) were received as output from Génome Québec. For each primer pair dataset, reads from the raw sequencing data were trimmed using *Cutadapt* (version 2.8) tool implemented in Unix Shell (version 4.4.19; Ubuntu version 18.04). For data pre-processing, the DADA2 pipeline (version 1.16.0; Callahan et al.^87^) was used to generate 16S rRNA (V3-V4) amplicon sequence variants (ASVs). Briefly, forward and reverse reads were trimmed, filtered and truncated based on their quality scores. The error model was calculated for forward and reverse reads. After denoising and merging, chimeric sequences (bimeras) were removed from the datasets by following the consensus method as implemented in DADA2. All subsequent analyses were performed within the R environment (R version 4.0.3, R Core Team^88^). The final table obtained consisted of a tabulation of number of occurrences of non-chimeric ASVs in each sample. Taxonomy assignments of representative ASVs were performed using the naïve Bayesian classifier method with the latest RDP 16 database. Sequences (4.8% of reads) attributed to Archaea or unclassified at phylum level were removed from the dataset. Raw datasets analyzed during the current study are publicly available on NCBI Sequence Read Archive (PRJNA773369).

### Statistical analysis

Bacterial community analysis were performed in the R environment using the *phyloseq*, *microbiomeSeq, microbiomeMarker* and *vegan* packages^89–92^. Alpha diversity indices, including the Shannon index, Pielou's evenness and richness estimator, were calculated using Wilcoxon/Kruskal–Wallis test between groups. Beta diversity between sample groups was determined based on the UniFrac distance and visualized by principal coordinates analysis (PCoA). Heatmaps were performed based on the relative abundance and constructed with the 30 most abundant genera. Bacterial biota composition differences among sites were studied using multivariate analysis of variance with permutation (PERMANOVA) with 9999 permutations. Permutation multivariate analysis of dispersion (PERMDISP) was also conducted with the function betadisper and permutest in order to test for homogeneity of multivariate dispersions (i.e., deviations from centroids) among sampling sites. The linear discriminant analysis (LDA) identifies the effect size (LEfSe) with which these taxa differentiate the samples. The threshold on the logarithmic score of LDA analysis was set to 2.0. Comparative metagenomic functional composition was predicted from the latest Kyoto Encyclopedia of Genes and Genomes (KEGG) database^93^ using a recently developed online tool Piphillin (http://secondgenome.com/Piphillin)^45,94^. The differential abundance analysis of gene abundance data was completed with the online tool MicrobiomeAnalyst^95^.

## Supplementary Information

Supplementary Information 1.

Supplementary Information 2.

## Abbreviations

*A. atra*: *Aulacomya atra*
*M. platensis*: *Mytilus platensis*
cmDNA: Circulating microbiome DNA, biodiversity
PCoA: Principal Coordinate Analysis
ASVs: Amplicon sequence variants
PERMANOVA: Multivariate analysis of variance with permutation
LEfSe: Linear discriminant analysis effect size
SOB: Sulphur-oxidizing bacteria
