# Supplementary material for: Species- and site-specific circulating bacterial DNA in Subantarctic sentinel mussels Aulacomya atra and Mytilus platensis
Source: Sci Rep. 2022 Jun 10;12:9547. doi: 10.1038/s41598-022-13774-1 (PMC9184546; doi:10.1038/s41598-022-13774-1)
Supplement: Supplementary file 1 — Supplementary Information 1. [file 41598_2022_13774_MOESM1_ESM.pdf]

**Supplementary material**

# Species- and site-specific circulating bacterial DNA in Subantarctic sentinel mussels *Aulacomya atra* and *Mytilus platensis*

Sophia Ferchiou[1], France Caza[1], Richard Villemur[1], Stéphane Betoulle[2], and Yves St-Pierre[1].

1) INRS-Centre Armand-Frappier Santé Technologie, 531 Boul. des Prairies, Laval, QC, Canada, H7V 1B7

2) Université Reims Champagne-Ardenne, UMR-I 02 SEBIO Stress environnementaux et Biosurveillance des milieux aquatiques, Campus Moulin de la Housse, 51687 Reims, France.

# Supp. Figure 1

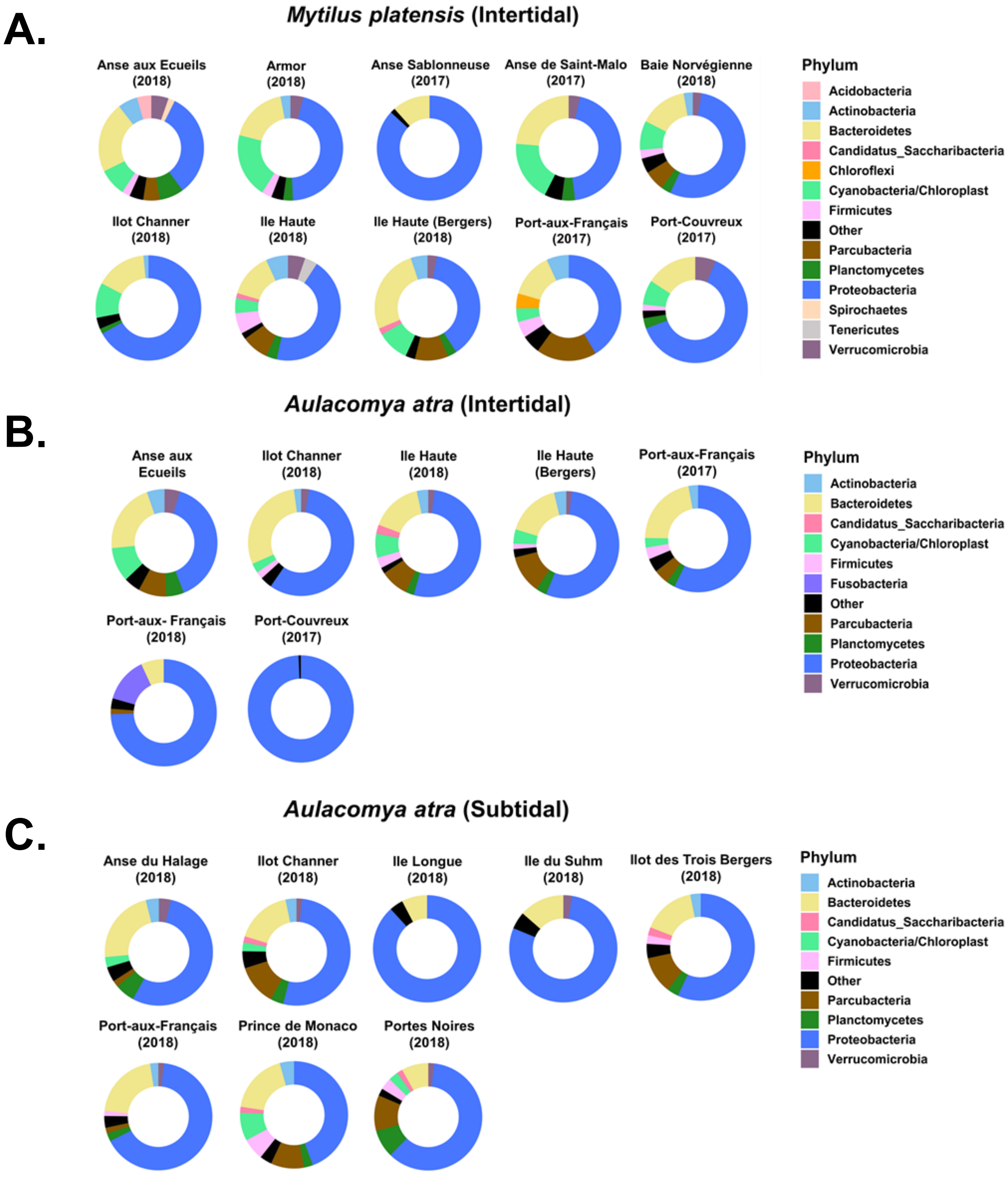


**Supp. Figure S1: Pie charts summarizing the phylum-level microbiota composition of the hemolymph of *Mytilus platensis* and *Aulacomya atra* in intertidal sites (A-B) and (C) of *Aulacomya atra* in subtidal sites during 2017 and 2018.** Phylum with a relative abundance of ≤ 1.5% are represented as "Other".

# Supp. Figure 2

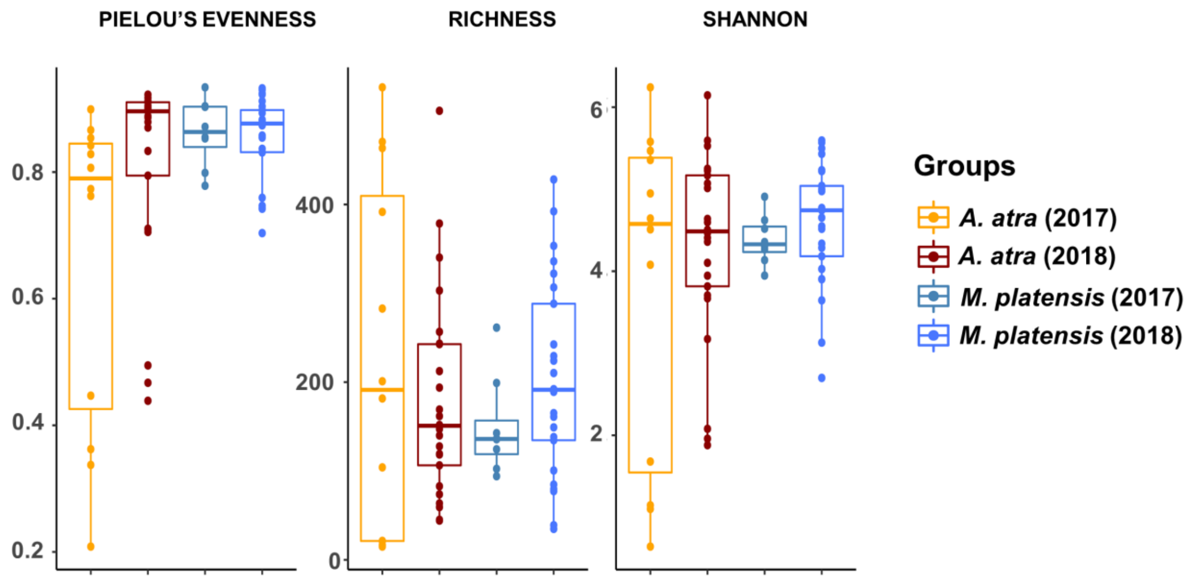


**Supp. Figure S2: Box plots of alpha diversity of *Aulacomya atra* and *Mytilus platensis* hemolymph microbiota in mixed mussel beds in 2017 and 2018.** Species evenness, observed richness and Shannon diversity indexes were calculated for each groups. No significant differences ($p < 0.05$) were observed between groups.

# Supp. Figure 3

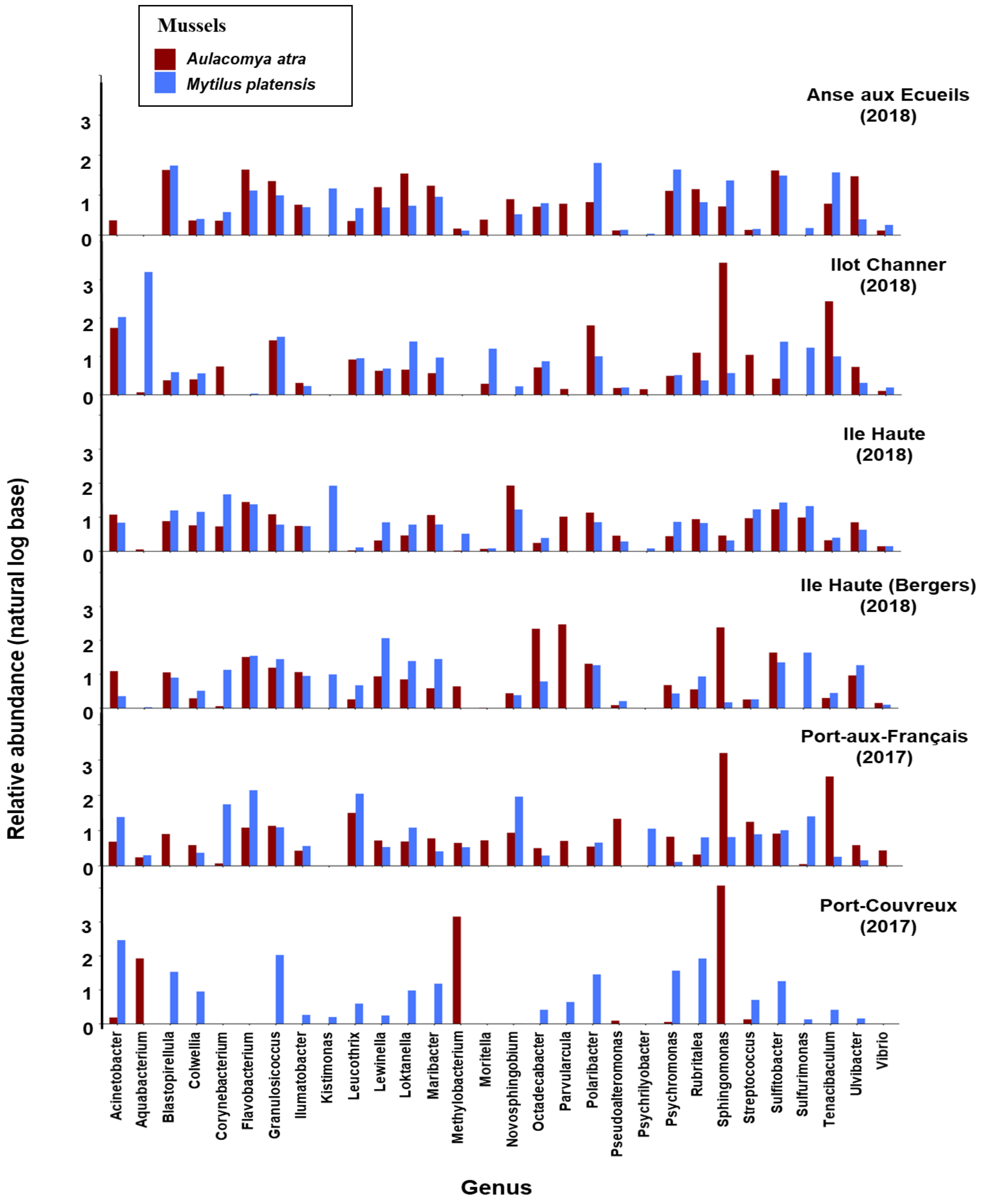


**Supp. Figure 3: Bar graphs showing the relative abundance (natural logarithm base) of the top 30 bacterial genera of circulating microbiota in mixed mussel beds.** All samples were collected in 2017 and 2018. Red color represents *Aulacomya atra* and blue color represents *Mytilus platensis* specimens.
